# Survey of Infections Transmissible Between Baboons and Humans, Cape Town, South Africa

**DOI:** 10.3201/eid1802.111309

**Published:** 2012-02

**Authors:** Julian A. Drewe, M. Justin O’Riain, Esme Beamish, Hamish Currie, Sven Parsons

**Affiliations:** Royal Veterinary College, London, UK (J.A. Drewe);; University of Cape Town, Cape Town, South Africa (M.J. O’Riain, E. Beamish);; Alphen Veterinary Hospital, Cape Town (H. Currie);; Stellenbosch University, Cape Town (S. Parsons)

**Keywords:** animals, wild, communicable diseases, infection, *Papio ursinus*, public health, zoonoses, anthroponoses, Cape Peninsula, South Africa

## Abstract

Baboons on South Africa’s Cape Peninsula come in frequent contact with humans. To determine potential health risks for both species, we screened 27 baboons from 5 troops for 10 infections. Most (56%) baboons had antibodies reactive or cross-reactive to human viruses. Spatial overlap between these species poses low but potential health risks.

The Cape Peninsula in South Africa is home to many species of wildlife, including ≈470 chacma baboons (*Papio ursinus*), which are a major tourist attraction and source of chronic conflict for local residents. Urban and agricultural land transformation has encroached markedly on the preferred natural habitat of baboons (*1*), and the 16 remaining troops on the Peninsula have been forced into marginal areas and are geographically isolated from all other baboon populations (Figure 1). The loss of preferred habitat, coupled with expanding numbers and a preference for high caloric food items, results in baboons entering residential areas daily to raid dustbins (garbage containers), enter homes, and attack humans in an effort to secure human-derived food (Figure 2).

**Figure 1:**
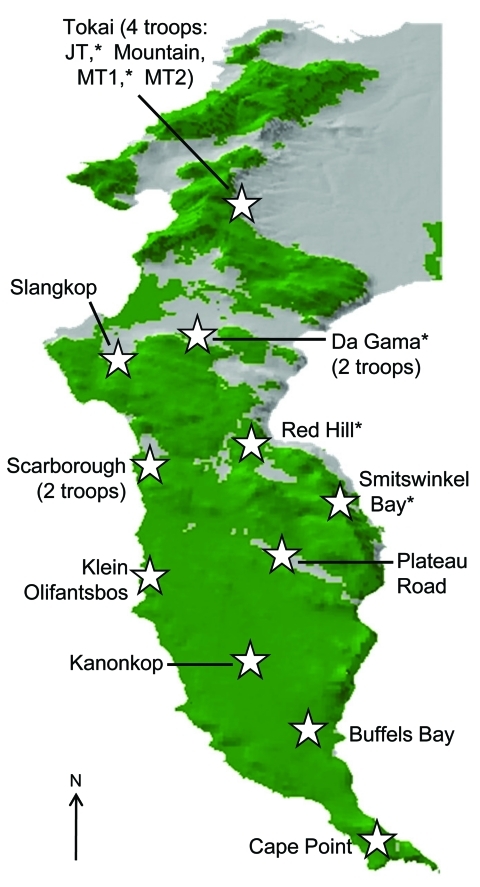
Cape Peninsula in South Africa, showing position and name of the different regions that have baboon troops. Baboons were sampled from those regions denoted by an asterisk. Green denotes natural land, and gray shows the current extent of urban and agricultural land on the Peninsula.

**Figure 2:**
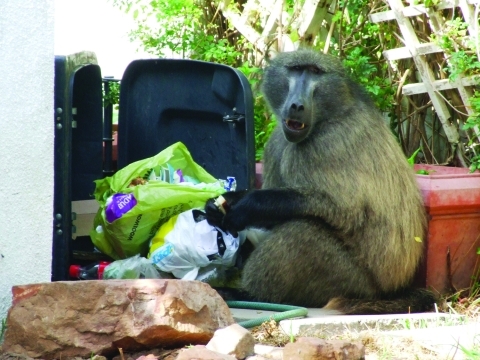
Baboon raiding a dustbin in the residential suburbs of Cape Town, South Africa.

The close contact between baboons and humans results in a high potential for the transmission of infectious diseases (*2*), from baboons to humans (zoonoses) and from humans to baboons (anthroponoses). Globally, disease transmission between humans and wildlife is occurring at an increasing rate, posing a substantial global threat to public health and biodiversity conservation (*3**,**4*). Although a study of baboon parasites in Kenya found none directly attributable to exposure to humans (*5*), the human parasite *Trichuris trichiura* has recently been identified in the Cape Peninsula baboon population; this finding represents the first evidence of likely anthroponotic infection of baboons (*6*). Diseases such as measles and tuberculosis are highly prevalent among the local human population (*7*) and have the potential to pass to baboons. The risks for infectious disease transmission between baboons and humans remain unclear. The aim of this study was to determine which diseases are currently present in the Cape Peninsula baboon population to inform decisions relating to baboon management, welfare and conservation, and the health risk to local humans and baboons. Ethical approval was gained from the Royal Veterinary College Ethics and Welfare Committee.

## The Study

Twenty-seven baboons (15 male, 12 female) from 5 troops were screened for 10 zoonotic infections in April 2011. A nonstratified power analysis indicated that this sample would provide >95% confidence of detecting infections if they were present at a prevalence of ≥10%. Pathogens were chosen for screening according to a literature review of infections in primates of potentially serious anthroponotic or zoonotic risk. Older animals were preferentially sampled because these were thought most likely to have been exposed to diseases. Fourteen adult baboons (7 males >7 years of age, 7 females >5 years of age), 7 subadult baboons (2 males 5–7 years of age, 5 females 4–5 years of age), and 6 juvenile baboons (6 males <5 years of age) were sampled. Nonrandom sampling was done to increase the chances of detecting diseases, if present, and was considered appropriate because the aim of this study was to determine presence or absence of infection, not prevalence of infection.

Baboons were individually trapped in cages and anesthetized for blood sampling. Samples of feces from each baboon were collected from the cage floor. After reversal of anesthesia and a suitable recovery period, the baboons were released in sight of their troop.

Automated enzyme linked fluorescent assays (Vidas; bioMérieux, Marcy l’Etoile, France) were used to test for antibodies against measles, hepatitis A virus (HAV), cytomegalovirus (CMV), and Epstein-Barr virus (Table 1). The manufacturer’s positive controls (human serum specimens containing IgG) were used. A serum neutralization test was used to screen samples for poliovirus antibodies. An interferon-gamma release assay for tuberculosis was conducted by using the QuantiFERON-TB Gold In-Tube test (Cellestis, Carnegie, Australia). This assay has been used previously for detection of *Mycobacterium tuberculosis* infection in chacma baboons (*8*). Test results were interpreted according to the manufacturer’s criteria for human patients. Feces samples were stored at 5°C for up to 24 h before being cultured for *Salmonella* spp., *Shigella* spp.*, Yersinia* spp., and *Campylobacter* spp. by using standard techniques (*9*).

**Table 1:** Results of diagnostic tests for exposure to 10 infectious diseases in 27 wild baboons, Cape Peninsula , South Africa, April 2011*

Infection	Diagnostic test	No. (%) baboons testing positive
CMV	Anti-CMV IgG ELFA	9 (33)
HAV	Anti-HAV total immunoglobulins ELFA	8 (30)
EBV	Anti-EBV early and nuclear antigens IgG ELFA	5 (19)
Measles virus	Anti-measles virus IgG ELFA	0
Polio virus	Serum neutralisation test	0
Tuberculosis	Whole blood gamma interferon test	0
*Salmonella* spp.	Fecal culture†	0
*Shigella* spp.	Fecal culture†	0
*Yersinia* spp.	Fecal culture†	0
*Camplyobacter* spp.	Fecal culture†	0

*CMV, cytomegalovirus; ELFA, enzyme-linked fluorescent assay; HAV, hepatitis A virus; EBV, Epstein-Barr virus. †Single fecal cultures performed on samples from 21 baboons only.

Results are shown in Table 1. Fifteen (56%) baboons had antibodies reactive or cross-reactive to at least 1 human virus: CMV, HAV, and Epstein-Barr virus. Seven (26%) baboons had antibodies reactive or cross-reactive to 2 of these viruses. Baboons in every troop were positive for at least 1 viral infection, but considerable variation was found among troops (Table 2). One troop (Da Gama) showed a higher than average rate of exposure to HAV; 6 (75%) of 8 of the HAV antibody-positive baboons were in this 1 troop, despite this troop’s representing just 7 (26%) of the 27 baboons in the sample. All 3 baboons sampled from another troop (Red Hill) had antibodies against CMV (Table 2). No pathogenic bacteria were found. Because intermittent shedding of fecal pathogens means that sampling animals on a single occasion may miss cases of infection (*10*), negative fecal culture results should not be considered definitive.

**Table 2:** Distribution of antibody-positive baboons by troop, Cape Peninsula , South Africa, April 2011*

Baboon troop	Predominant human habitat type	No. baboons tested	No. (%) CMV positive	No. (%) HAV positive	No. (%) EBV positive
Red Hill	Urban residential	3	3 (100)	1 (33)	1 (33)
Da Gama	Urban residential	7	3 (43)	6 (86)	1 (14)
Smitswinkel Bay	Scenic tourist route	3	1 (33)	0	1 (33)
Tokai JT	Forest plantation	6	0	1 (17)	2 (33)
Tokai MT1	Forest plantation	8	2 (25)	0	0
Totals		27	9	8	5

*The locations of each baboon troop are indicated in Figure 1. CMV, cytomegalovirus; HAV, hepatitis A virus; EBV, Epstein-Barr virus.

## Conclusions

This study provides evidence of the potential for cross-species trafficking of select pathogens. Widespread evidence of reactive or cross-reactive humoral immune responses to human pathogens was found in wild baboons. The detection of antibodies reactive or cross-reactive to HAV in 30% of baboons tested is a potential cause for concern. Because HAV is spread by the fecal–oral route, many opportunities might exist for direct and indirect transmission between baboons and humans; e.g., baboons frequent picnic sites and enter houses and cars in search of food. The frequency with which such contacts result in transmission of HAV should be investigated because of the potentially fatal consequences of human infection with HAV, particularly for immunocompromised persons such as those co-infected with HIV. Furthermore, as pathogens pass back and forth across species lines, the potential for changes in pathogenicity and host specificity exists, which can result in serious adverse effects on human and wildlife health.

The considerable variation in virus immunity among baboon troops (Table 2) warrants further study. The difference was particularly pronounced in the 2 most sampled troops, in which HAV antibody prevalence varied from 0% (0/8 baboons in the Tokai MT1 troop, in a forest) to 86% (6/7 baboons in the Da Gama troop, in an urban area). Future work should target these groups for more extensive sampling (ideally, all baboons should be sampled) to more accurately determine the prevalence of infection and investigate risk factors for virus exposure. A suitable hypothesis for testing would be that zoonotic infection prevalence in baboons is positively correlated with the proportion of urban land in their habitat.

The results of this study suggest that baboons on the Cape Peninsula pose a low but potential risk for transmitting zoonoses and that they might be at risk from anthroponoses. The findings should not be interpreted as definitively showing baboon exposure to human viruses because the serologic tests did not distinguish between human and baboon variants of the viruses and some cross-reactivity may have occurred. Virus isolation would be needed to determine the virus types. Nonetheless, there is ample evidence that disease of human origin can be devastating for primate populations (*11**,**12*). Further research is required on the Cape Peninsula to quantify the incidence of infections in baboons and humans, to examine the variation in levels of infection among baboon troops, and to measure the frequency of contact between species. Estimating the probability of cross-species disease transmission is challenging (*13*), but this information would be of tremendous use in informing baboon management plans with the aim of reducing the risks for infectious disease in humans and baboons.

## References

[1] Hoffman TS, O’Riain MJ. The spatial ecology of chacma baboons (*Papio ursinus*) in a human-modified environment. Int J Primatol. 2011;32:308–28. 10.1007/s10764-010-9467-6

[2] Gillespie TR, Nunn CL, Leendertz FH. Integrative approaches to the study of primate infectious disease: implications for biodiversity conservation and global health. Am J Phys Anthropol. 2008;137:53–69. 10.1002/ajpa.20949 19003885

[3] Daszak P, Cunningham AA, Hyatt AD. Emerging infectious diseases of wildlife: threats to biodiversity and human health. Science. 2000;287:443–9. 10.1126/science.287.5452.443 10642539

[4] Jones KE, Patel NG, Levy MA, Storeygard A, Balk D, Gittleman JL, Global trends in emerging infectious diseases. Nature. 2008;451:990–3. 10.1038/nature06536 18288193 PMC5960580

[5] Hahn NE, Proulx D, Muruthi PM, Alberts S, Altmann J. Gastrointestinal parasites in free-ranging Kenyan baboons (*Papio cynocephalus* and *P. anubis*). Int J Primatol. 2003;24:271–9. 10.1023/A:1023092915171

[6] Ravasi DFC. Gastrointestinal parasite infections in chacma baboons (*Papio ursinus*) of the Cape Peninsula, South Africa: the influence of individual, group and anthropogenic factors [PhD thesis]. University of Cape Town, South Africa; 2009.

[7] World Health Organization. Global tuberculosis control: epidemiology, strategy and financing (WHO/HTM/TB/2009.411). 2009 [cited 2011 Jun 7] http://www.who.int/tb/publications/global_report/2009/pdf/chapter1.pdf.

[8] Parsons SDC, Gous TA, Warren RM, de Villiers C, Seier JV, van Helden PD. Detection of *Mycobacterium tuberculosis* infection in chacma baboons (*Papio ursinus*) using the QuantiFERON-TB Gold (In-Tube) assay. J Med Primatol. 2009;38:411–7. 10.1111/j.1600-0684.2009.00367.x 19627435

[9] Nizeyi JB, Innocent RB, Erume J, Kalema G, Cranfield MR, Graczyk TK. Campylobacteriosis, salmonellosis, and shigellosis in free-ranging human-habituated mountain gorillas of Uganda. J Wildl Dis. 2001;37:239–44. 11310873 10.7589/0090-3558-37.2.239

[10] Morner T. Miscellaneous bacterial infections. In: Williams E, Barker I, editors. Infectious diseases of wild mammals, 3rd ed. Ames (IA): Iowa State University Press; 2001. p. 487–513.

[11] Palacios G, Lowenstine LJ, Cranfield MR, Gilardi KVK, Spelman L, Lukasik-Braum M, Human metapneumovirus infection in wild mountain gorillas, Rwanda. Emerg Infect Dis. 2011;17:711–3. 21470468 10.3201/eid1704.100883 PMC3377396

[12] Köndgen S, Kühl H, N’Goran PK, Walsh PD, Schenk S, Ernst N, Pandemic human viruses cause decline of endangered great apes. Curr Biol. 2008;18:260–4. 10.1016/j.cub.2008.01.012 18222690

[13] Lloyd-Smith JO, George D, Pepin KM, Pitzer VE, Pulliam JRC, Dobson AP, Epidemic dynamics at the human-animal interface. Science. 2009;326:1362–7. 10.1126/science.1177345 19965751 PMC3891603

